# Systematic review and meta-analysis of omalizumab for IgE-mediated food allergy in children and young adults

**DOI:** 10.3389/fimmu.2025.1690650

**Published:** 2025-10-23

**Authors:** Ya Zhang, Bin Liang, Liang Tian, Bin Chen, Shanshan Wu

**Affiliations:** ^1^ Department of Pediatrics, Xichang People’s Hospital, Sichuan, China; ^2^ Department of Pediatrics, Sichuan Provincial People’s Hospital, School of Medicine, University of Electronic Science and Technology of China, Chengdu, China; ^3^ Department of Pediatrics, West China Second University Hospital, Sichuan University, Chengdu, China

**Keywords:** omalizumab, food allergy, allergen immunotherapy, adverse reaction, children, young adults

## Abstract

**Objectives:**

This meta-analysis aims to evaluate the efficacy of omalizumab (as monotherapy or combined with OIT) in achieving target maintenance dose (TMD), and its safety profile in terms of treatment-emergent adverse events (TEAEs) and epinephrine use, in children and young adults with IgE-mediated food allergy.

**Methods:**

A comprehensive literature search was conducted across PubMed, Embase, Cochrane Library, and Web of Science databases to identify relevant randomized controlled trials (RCTs) and controlled clinical trials (CCTs). Eligible studies compared omalizumab-based interventions (monotherapy or OIT combination) with control strategies (placebo, placebo plus OIT, or strict allergen avoidance) in children and adolescents with IgE-mediated food allergy. The primary efficacy endpoint was the achievement of a target maintenance dose (TMD), defined as the maximum allergen dose tolerated without dose-limiting symptoms. Safety outcomes included the incidence of treatment-emergent adverse events (TEAEs) and the requirement for epinephrine administration. Data synthesis employed random-effects models to calculate pooled risk ratios (RRs) with 95% confidence intervals (CIs).

**Results:**

Eight studies (n = 734 participants) met the inclusion criteria, comprising 7 RCTs and 1 CCT. Pooled analysis demonstrated that omalizumab-based therapy significantly increased the likelihood of achieving a clinically meaningful TMD compared to control interventions (RR = 3.07, 95% CI: 1.42–6.62; p < 0.001), with consistent efficacy observed across subgroups of multiple food allergies, peanut allergy. With respect to safety, no statistically significant difference was noted in the overall incidence of TEAEs between the omalizumab and control groups (RR = 1.02, 95% CI: 0.74–1.41; p = 0.889). Similarly, the rate of epinephrine use during oral food challenges or treatment did not differ significantly between groups (RR = 0.59, 95% CI: 0.07–4.78; p = 0.099), though the wide confidence intervals indicate substantial uncertainty due to limited data.

**Conclusion:**

This meta-analysis provides robust evidence that omalizumab, either as monotherapy or in combination with OIT, significantly enhances allergen tolerance in children and young adults with IgE-mediated food allergy. Importantly, this therapeutic benefit is not accompanied by a significant increase in the overall burden of adverse events or epinephrine use relative to control strategies. Omalizumab thus represents a valuable therapeutic option for improving desensitization outcomes in this vulnerable population.

**Systematic review registration:**

https://www.crd.york.ac.uk/prospero/, identifier CRD420251087191.

## 1 Introduction

Food allergy (FA) has emerged as a critical public health challenge affecting children and adolescents globally, with escalating prevalence over recent decades. Current estimates indicate that approximately 5-10% of children worldwide suffer from immunoglobulin E (IgE)-mediated food allergies, with common triggers including peanut, tree nuts, milk, egg, soy, wheat, fish, and shellfish (1–3). Regional variations exist, yet studies consistently report increasing incidence and healthcare utilization related to FA (4, 5). Beyond immediate physical morbidity—ranging from urticaria and gastrointestinal distress to life-threatening anaphylaxis—pediatric FA imposes profound psychosocial burdens. Affected children experience heightened anxiety (6), social isolation (7), bullying (8), and significantly reduced health-related quality of life (HRQL), impacting both the child and their caregivers. The constant threat of accidental exposure necessitates strict avoidance, creating significant limitations on daily activities (e.g., eating outside the home, attending school) and contributing to substantial economic costs through emergency department visits, hospitalizations, and lost productivity (9, 10). This pervasive impact underscores the urgent need for effective disease-modifying therapies.

The cornerstone of FA management remains strict allergen avoidance and prompt administration of rescue medications, primarily intramuscular epinephrine, during reactions (11). While essential, avoidance is inherently imperfect, leading to unpredictable and potentially severe reactions upon accidental exposure (12). Oral Immunotherapy (OIT) represents an active treatment approach aimed at inducing desensitization through the gradual ingestion of increasing allergen doses. While numerous studies demonstrate OIT’s efficacy in raising reaction thresholds for specific allergens like peanut and egg, significant limitations persist (13–15). Treatment-emergent adverse events (TEAEs) are common, including oral pruritus, gastrointestinal symptoms, and systemic reactions requiring epinephrine, contributing to high dropout rates in real-world settings (16, 17). Achieving sustained unresponsiveness (SU) after discontinuation remains elusive for a substantial proportion of patients, and long-term adherence is challenging (18, 19). Furthermore, OIT is time-intensive, requires specialized clinical settings, and is not universally effective across all allergens or individuals (20). These limitations highlight the critical unmet need for safer, more effective, and broadly applicable therapeutic strategies for pediatric FA.

Omalizumab, a recombinant humanized monoclonal anti-IgE antibody, offers a distinct immunomodulatory approach. It binds free IgE, preventing its interaction with the high-affinity IgE receptor (FcϵRI) on mast cells and basophils, thereby reducing their reactivity and the potential for degranulation upon allergen encounter (21). This mechanism positions omalizumab uniquely as both a potential monotherapy and a facilitator for other treatments like OIT. Preclinical and early clinical data suggested omalizumab could modulate the allergic response threshold (22). Recent phase 3 trials provide compelling evidence: the pivotal OUTMATCH study demonstrated omalizumab significantly increased the reaction threshold to peanut and multiple other common allergens in multi-food allergic children and adolescents compared to placebo (23). Crucially, the PROTECT trial confirmed these findings specifically in peanut-allergic children aged 1–17 years, showing significantly higher peanut tolerance thresholds and improved quality of life measures in the omalizumab group (24). Its recent FDA approval for IgE-mediated food allergy based on these trials marks a significant advancement (25). As monotherapy, omalizumab offers the potential advantage of multi-food allergen coverage without the need for daily allergen ingestion and its associated AEs. When combined with OIT, omalizumab has been shown to enhance safety (reducing reaction rates and severity) and efficacy (enabling faster up-dosing and higher maintenance doses) (26, 27). However, while individual RCTs demonstrate efficacy, a comprehensive quantitative synthesis focusing specifically on the pediatric and adolescent population is needed to definitively establish the magnitude of benefit and safety profile across diverse studies. Therefore, this meta-analysis aims to rigorously evaluate the efficacy (focusing on TMD achievement) and safety (including TEAEs and epinephrine use) of omalizumab, either as monotherapy or in combination with OIT, for food allergy specifically in children and adolescents. Unlike previous meta-analyses, this study focuses exclusively on pediatric and young adult populations, includes recent Phase 3 trials, and provides updated safety and efficacy estimates. By pooling data from all available randomized controlled trials, this analysis seeks to provide a higher level of evidence to guide clinical practice and inform future research.

## 2 Methods

This meta-analysis was conducted in accordance with the Preferred Reporting Items for Systematic Reviews and Meta-Analyses (PRISMA) guidelines (28) and was prospectively registered in PROSPERO (CRD420251087191) prior to data extraction. The primary objective was to evaluate the efficacy and safety of omalizumab (either as monotherapy or in combination with OIT) compared to control interventions for IgE-mediated food allergy in children and young adults.

### 2.1 Search strategy and study selection

A systematic literature search was performed across PubMed, Embase, Cochrane Central Register of Controlled Trials (CENTRAL), and Web of Science from database inception to June 30, 2025. Search terms included: “omalizumab,” “Xolair,” “anti-IgE,” “food allergy,” “oral immunotherapy,” “randomized controlled trial,” and “RCT.” Only English-language studies were included. Additional relevant studies were identified through manual searches of reference lists of included trials and related systematic reviews. Conference abstracts were screened but excluded if insufficient data were provided.

### 2.2 Eligibility criteria

RCTs and CCTs involving participants aged 1–26 years with a clinical diagnosis of IgE-mediated food allergy (either single or multiple allergen sensitization) were included, provided they compared omalizumab (as monotherapy or in combination with OIT) to control groups, which included placebo, placebo plus OIT, or strict allergen avoidance. Non-randomized studies, RCTs/CCTs without extractable data, trials excluding pediatric populations, preclinical studies, retrospective studies, case reports, reviews, consensus reports, and studies with insufficient outcome data.

### 2.3 Study selection process

**Figure 1:**
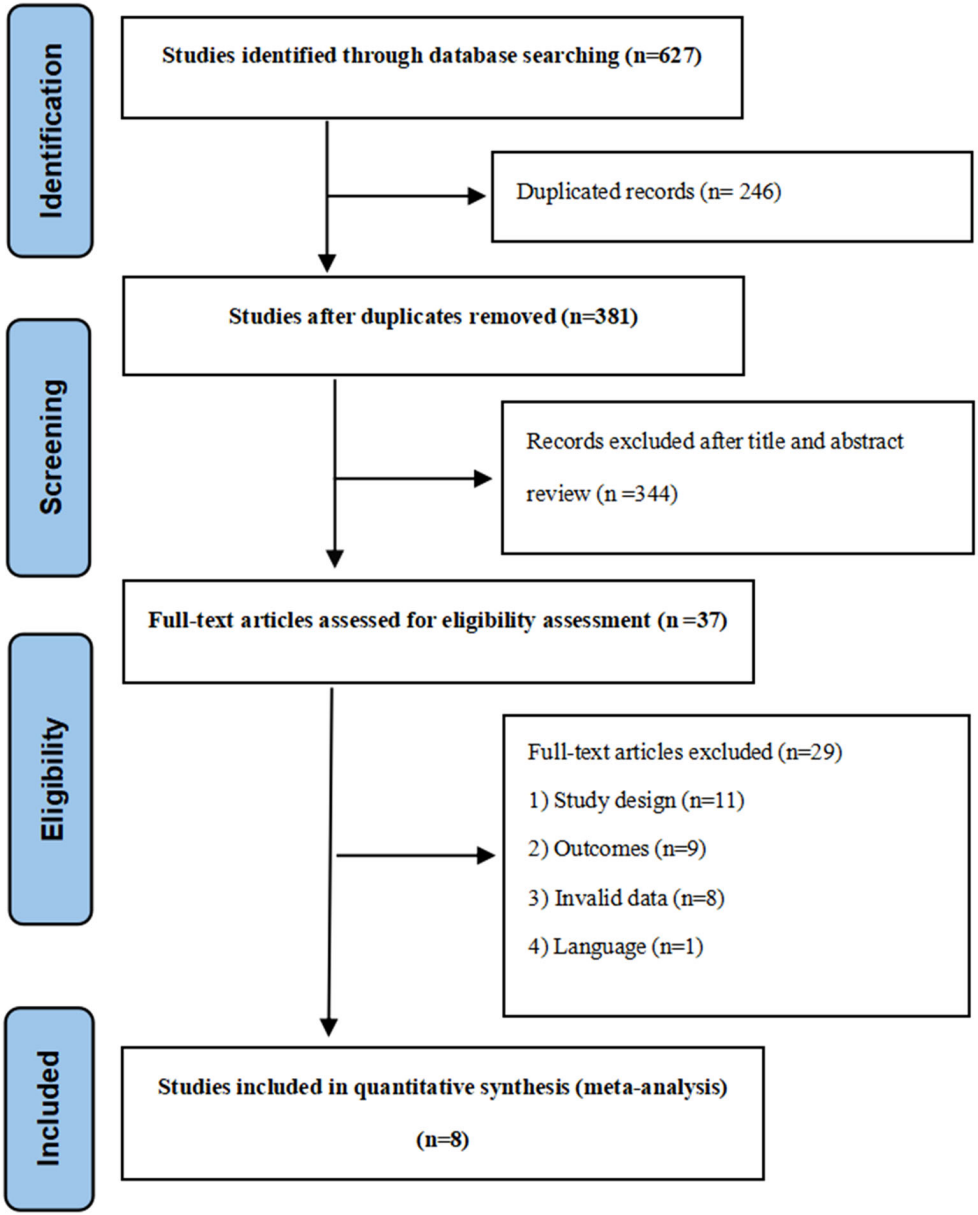
Flow chart of the literature search.

Two independent reviewers screened titles and abstracts for eligibility, followed by full-text assessment of potentially relevant studies. Disagreements were resolved through discussion or consultation with a third reviewer. The selection process was documented using a PRISMA flow diagram (**Figure 1**).

### 2.4 Data extraction

Data extraction was performed independently by two reviewers using a pre-piloted electronic form. Extracted information included: Study characteristics: Author(s), publication year, study location, design (RCT/CCT), sample size, age range of participants, and specific food allergens targeted. Intervention details: Omalizumab dosing regimen (fixed dose, weight/IgE-based dose, or unspecified), treatment duration, OIT protocols (where applicable), and control group interventions (placebo, placebo + OIT, or strict avoidance). Outcomes: Data on target maintenance dose (TMD), defined as the highest tolerated dose of food allergen without dose-limiting symptoms, as well as TEAEs and epinephrine use during treatment or oral food challenges.

### 2.5 Quality assessment

Methodological quality of included studies was assessed independently by two reviewers, with Cochrane Collaboration’s Risk of Bias tool (RoB 2) (29) applied specifically to RCTs and Cochrane ACROBAT-NRS tool (30) used for CCTs.

For RCTs evaluated via RoB 2, the assessment focused on five core domains that address trial-specific bias risks: (i) Bias arising from the randomization process (to evaluate the integrity of random sequence generation and allocation concealment); (ii) Bias due to deviations from intended interventions (to assess protocol adherence, including deviations in intervention delivery and participant compliance); (iii) Bias due to missing outcome data (to examine the quantity, reason, and handling of missing data and their potential impact on results); (iv) Bias in measurement of outcomes (to judge the validity of outcome measurement methods, including blinding of assessors and reliability of measurement tools); (v) Bias in selection of the reported result (to identify selective reporting, such as discrepancies between pre-specified outcomes in protocols and reported outcomes in manuscripts).

For CCTs assessed using the ACROBAT-NRS tool, the evaluation targeted five domains tailored to non-randomized study designs: (i) Bias arising from the selection of participants into the study (to evaluate how participants were assigned to intervention and control groups, and potential confounding); (ii) Bias due to deviations from intended interventions (to assess consistency in intervention implementation and whether deviations differed between groups); (iii) Bias due to missing outcome data (to analyze the extent, cause, and handling of missing data, and their influence on effect estimates); (iv) Bias in measurement of outcomes (to appraise the objectivity of outcome assessment, including whether assessors were blinded to group allocation); (v) Bias in selection of the reported result (to check for selective reporting of outcomes, analyses, or time points).

For both tools, judgments for each individual domain were categorized as “Low Risk,” “Some Concerns,” or “High Risk” based on predefined criteria in the respective Cochrane tools. An overall methodological quality assessment for each included study was finalized through consensus between the two reviewers to resolve any initial discrepancies.

### 2.6 Data synthesis and statistical analysis

All analyses used the DerSimonian and Laird random-effects model to account for anticipated clinical and methodological heterogeneity across studies. For studies reporting TMD as a continuous variable (e.g., mean cumulative tolerated dose in mg of protein with standard deviation), the standardized mean difference (SMD) with 95% confidence intervals (CIs) was calculated to enable pooling across allergens and measurement scales. For studies reporting TMD as a dichotomous outcome (e.g., proportion of participants achieving a protocol-defined threshold such as ≥300 mg peanut protein or equivalent), the risk ratio (RR) with 95% CI was calculated, comparing omalizumab-based interventions to controls. The incidence of TEAEs and epinephrine use was analyzed using pooled RRs with 95% CIs.

Heterogeneity was quantified using the I² statistic and Cochran’s Q-test, with I² <25% indicating low heterogeneity, 25–50% moderate heterogeneity, and >50% substantial heterogeneity; a p-value <0.10 for the Q-test was considered indicative of significant heterogeneity. A continuity correction of 0.5 was applied to contingency tables with zero cells. Intention-to-treat (ITT) data were used for primary efficacy analyses where available; otherwise, complete case analysis was performed. Despite variability in TMD thresholds, all definitions reflected clinically meaningful desensitization goals. We conducted subgroup analyses by threshold to ensure robustness and clinical interpretability. Subgroup analyses were pre-specified based on clinical relevance: treatment duration (<30 vs ≥30 weeks) to assess short- vs longer-term effects; allergen type (multiple, peanut, milk) to evaluate consistency across sensitization profiles; and we conducted subgroup analyses stratified by TMD thresholds (e.g., ≥300 mg, ≥2 g) to assess consistency across definitions.

Sensitivity analyses were conducted using an influence analysis approach, sequentially excluding each study to assess its impact on overall effect estimates. Sensitivity analyses were conducted excluding the non-randomized CCT to assess its influence on pooled estimates. Publication bias was evaluated visually using funnel plots (if ≥10 studies were included) and statistically via Egger’s linear regression test. All analyses were performed using STATA version 15.1. Statistical significance was defined as a two-tailed p-value <0.05. The certainty of evidence for critical outcomes (TMD, TEAEs, epinephrine use) was evaluated using the Grading of Recommendations Assessment, Development and Evaluation (GRADE) framework by two independent reviewers.

## 3 Results

### 3.1 Study selection and characteristics

The systematic literature search identified 627 records. After title/abstract screening and full-text assessment, eight studies met the inclusion criteria, comprising seven RCTs (23, 31–36) and one CCT (37). Study characteristics are summarized in **Table 1**.

**Table 1:** Baseline characteristics and primary results of included trials.

Study	Country	Design	Sample size(male)	Age (years)	Type of allergy	Intervention	OMA does	Comparison	Duration (weeks)	Results
Andorf et al. (2018) (31)	USA	Phase 2 RCT	48 (24)	4-15	Multiple	OMA+OIT	–	Placebo+OIT	36	TMD, TEAEs
Andorf et al. (2019) (38)	USA	Phase 2 RCT	60 (37)	5-22	Multiple	OMA+OIT	300mg	Placebo+OIT	36	TMD, TEAEs, Epinephrine
MacGinnitie et al. (2017) (39)	USA	Phase 2 RCT	37 (22)	7-19	Peanut	OMA+OIT	250mg	Placebo+OIT	20	TMD, TEAEs
Mortz et al. (2024) (24)	Denmark	Phase 2 RCT	20 (12)	6-17	Multiple	OMA+OIT	–	Placebo+OIT	24	TMD, TEAEs
Sampson et al. (2011) (35)	USA	Phase 2 RCT	14 (7)	16-26	Peanut	OMA	0.016 mg/kg/IgE	Placebo	24	TMD, TEAEs
Takahashi et al. (2017) (37)	Japan	CCT	16 (11)	6-14	Cow's milk	OMA+OIT	1500 IU/mL/body weight	Avoidance	32	TMD, TEAEs
Wood et al. (2016) (40)	USA	Phase 3 RCT	57 (40)	8-15	Cow's milk	OMA+OIT	300mg	Placebo+OIT	128	TMD, TEAEs, Epinephrine
Wood et al. (2024) (41)	USA	Phase 3 RCT	177 (100)	1-17	Multiple	OMA+OIT	75-600mg	Placebo+OIT	16	TMD, TEAEs

CCT, controlled clinical trial; OMA, omalizumab; OIT, Oral immunotherapy; TMD, target maintenance dose; TEAEs, treatment-emergent adverse events.

Six studies were conducted in the USA, one in Denmark, and one in Japan. Five were Phase 2 RCTs, two were Phase 3 RCTs, and one was a CCT. Total sample sizes ranged from 14 to 177 participants, with participants aged 1–26 years. Four studies enrolled participants with multiple food allergies (≥2 allergens), two focused exclusively on peanut allergy, and two on cow’s milk allergy. One study evaluated omalizumab monotherapy, while seven investigated omalizumab combined with oral immunotherapy (OMA+OIT). Omalizumab dosing varied: three studies used fixed doses (75–375 mg), three employed weight/IgE-based dosing (0.016–0.44 mg/kg/IgE[IU/mL] per month), and two did not specify regimens. Six studies used placebo + OIT, one used placebo alone, and one used strict allergen avoidance. Treatment duration ranged from 16 to 128 weeks. All eight studies provided data on TMD and TEAEs; only two studies reported data on epinephrine use.

### 3.2 Quality assessment

**Figure 2:**
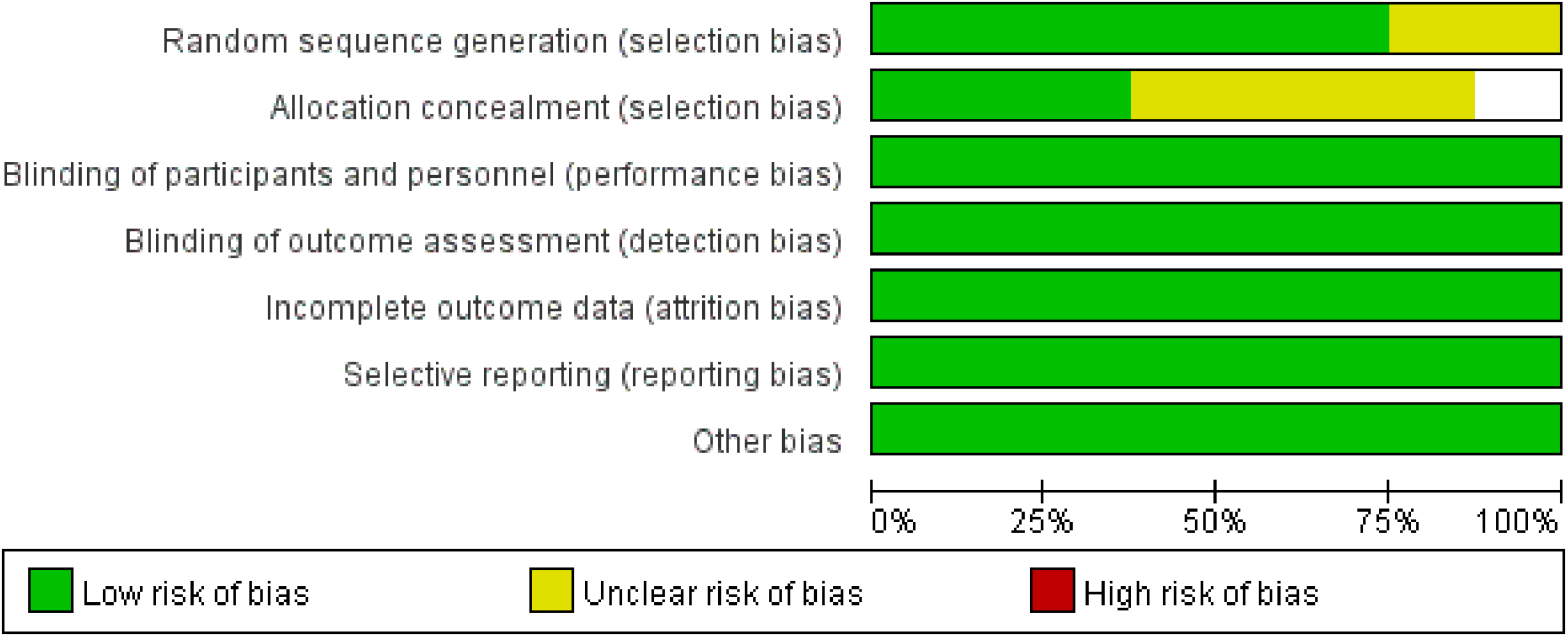
Risk of bias graph.

**Figure 3:**
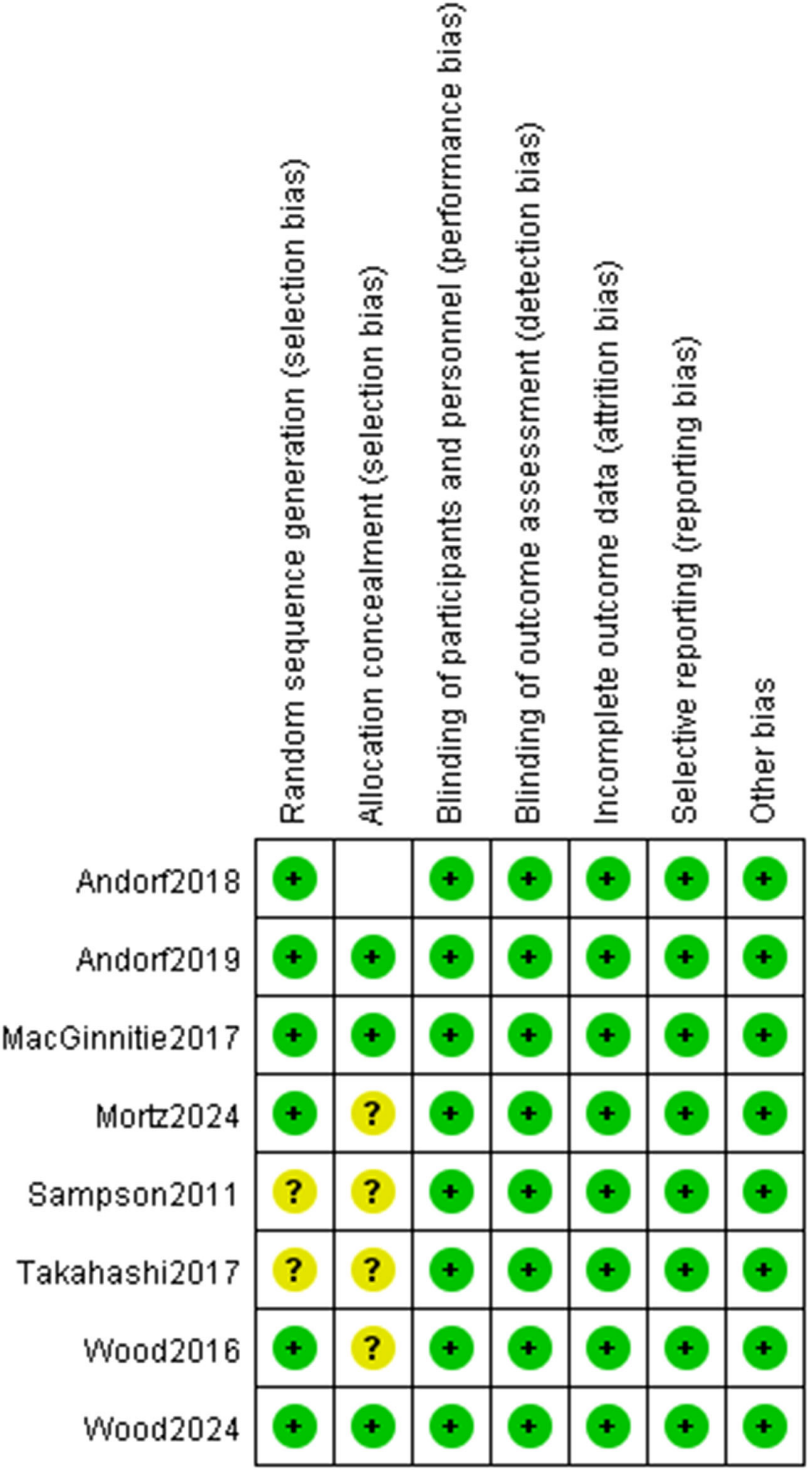
Risk of bias summary.

The methodological quality of included studies was assessed using Cochrane tools: seven RCTs with the RoB 2 tool, and one CCT with the ACROBAT-NRS tool (**Figures 2**, **3**). For RCTs, six had low risk of random sequence generation (e.g., computerized randomization) and five for allocation concealment (e.g., opaque envelopes); one RCT (38) had high performance bias (open-label design). Five RCTs had low detection bias (blinded outcome assessment for DBPCFC/laboratory indices), while two had some concerns (unreported SPT assessor blinding). All RCTs had low attrition (no/incomplete dropout, ITT analysis) and reporting bias (full prespecified outcome reporting). The CCT had high selection bias (caregiver-preference allocation, baseline imbalance) and detection bias (unblinded assessors), but low attrition/reporting bias. Six RCTs had low overall bias; the CCT’s high bias did not alter primary efficacy (TMD: RR = 3.22, p<0.001) per sensitivity analysis. Risk of bias assessment revealed that six RCTs had low overall risk, while one RCT had high performance bias due to open-label design. The single CCT had high selection and detection bias. Sensitivity analyses excluding the CCT did not alter the primary efficacy outcome, suggesting minimal bias impact on pooled estimates.

### 3.3 Efficacy outcomes

**Figure 4:**
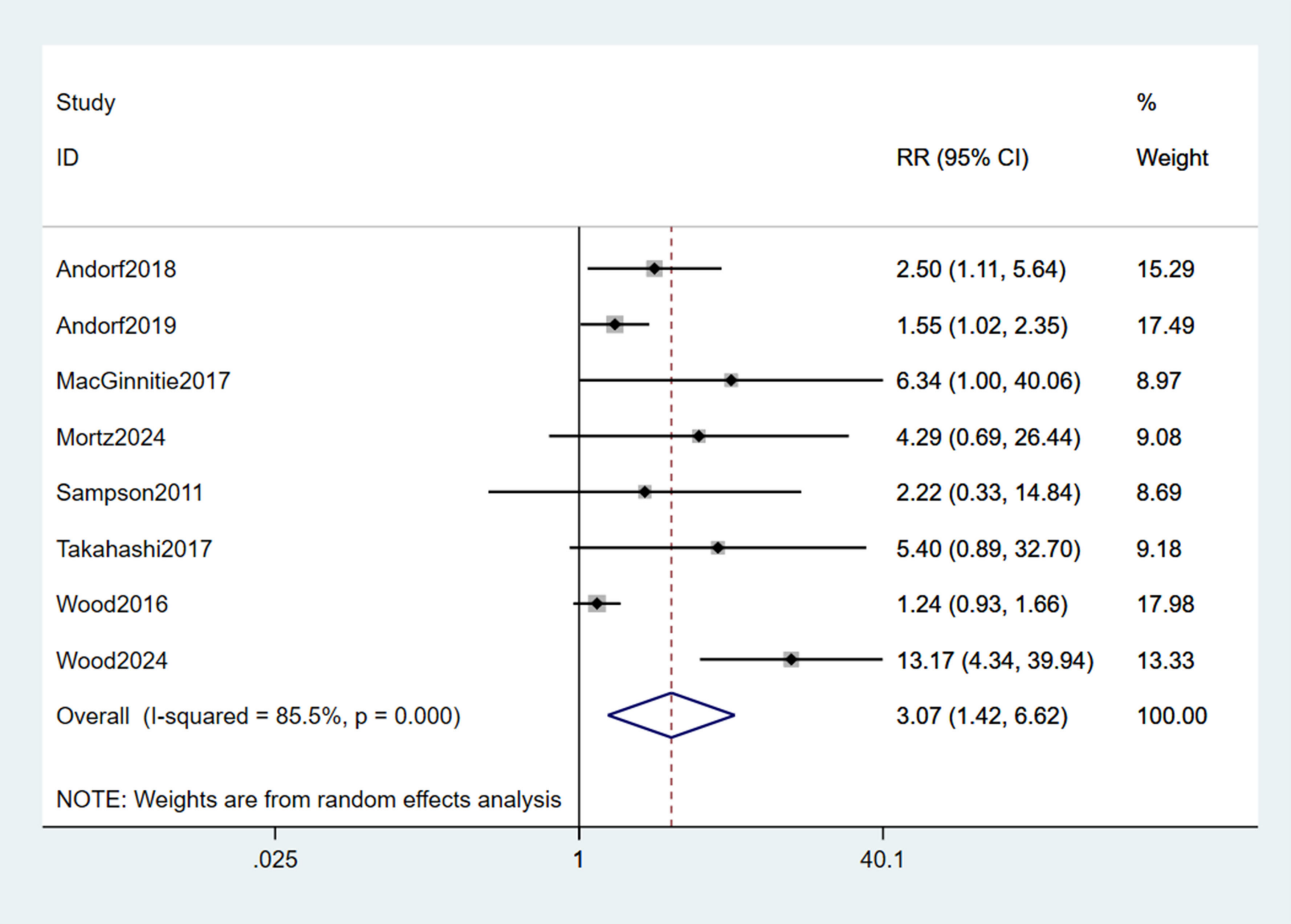
Forest plot of TMD.

A pooled analysis of the 8 included studies demonstrated that omalizumab-based interventions—whether administered as monotherapy or in combination with oral immunotherapy (OMA+OIT)—significantly increased the likelihood of achieving a clinically meaningful TMD compared to control strategies (placebo, placebo plus OIT, or strict allergen avoidance). The pooled RR for this effect was 3.07 (95% CI: 1.42–6.62; p < 0.001; **Figure 4**), indicating that children and young adults receiving omalizumab-based therapy were over three times more likely to reach the predefined TMD than those in control groups. However, substantial statistical heterogeneity was observed across studies (I² = 72%, p = 0.001), reflecting variability in intervention protocols, allergen types, and TMD definitions.

Subgroup analyses (**Table 2**) confirmed the consistent efficacy of omalizumab-based therapy across various subgroups. Stratified analyses confirmed the consistent efficacy of omalizumab-based therapy across different allergen-specific subgroups. In studies focusing on participants with sensitization to ≥2 food allergens, omalizumab-based therapy significantly improved TMD achievement (RR = 3.60, 95% CI: 1.06–12.25; p < 0.001). This subgroup included 4 studies, with substantial heterogeneity (I² = 86.8%, p = 0.000), likely due to variations in the number and types of allergens targeted. For participants with isolated peanut allergy, the therapeutic benefit remained statistically significant (RR = 3.81, 95% CI: 1.02–14.32; p = 0.046). Two studies contributed to this subgroup, with no significant heterogeneity (I² = 0.0%, p = 0.416), indicating consistent effects across peanut-specific interventions. In the subgroup of participants with cow’s milk allergy, the RR was 2.12 (95% CI: 0.41–11.00; p = 0.37), which did not reach statistical significance. This finding may be attributed to the limited sample size (2 studies) and moderate heterogeneity (I² = 71.5%, p = 0.061) in this subgroup.

**Table 2:** Subgroup analyses of TMD.

Subgroup	Sample size	Risk ratios	95% confidence interval
Multiple allergy	305	3.60	1.06, 12.25
Peanut allergy	54	3.81	1.02, 14.32
Cow's milk allergy	73	2.12	0.41, 11.00
Duration <30 weeks	248	6.90	3.08-15.43
Duration ≥30 weeks	181	1.64	1.07, 2.50
Tolerated 2g of ≥2 allergens	285	3.22	0.91, 11.44
Tolerated 2g of ≥3 allergens	252	4.13	0.95, 17.86
Tolerated 2g of ≥4 allergens	49	2.87	1.22, 6.75
OMA monotherapy	14	2.22	0.33, 14.84
OMA+OIT	368	3.19	1.40, 7.28

(Sample size reflects total participants in each subgroup. Risk ratios (RR) with 95% CIs are reported; RR>1 indicates omalizumab-based therapy improves TMD achievement compared to controls).

Stratification by treatment duration (<30 weeks vs. ≥30 weeks) further clarified the efficacy profile of omalizumab-based therapy. The threshold of 30 weeks was chosen to distinguish between short-term desensitization and longer-term maintenance phases, reflecting typical OIT trial designs. In 4 studies with treatment durations less than 30 weeks, omalizumab-based therapy was strongly associated with increased TMD achievement (RR = 6.90, 95% CI: 3.08–15.43; p < 0.001). Minimal heterogeneity was observed in this subgroup (I² = 6.3%, p = 0.362), suggesting consistent short-term effects. For studies with treatment durations of 30 weeks or longer, the therapeutic effect remained statistically significant but was of smaller magnitude (RR = 1.64, 95% CI: 1.07–2.50; p = 0.023). Moderate heterogeneity was noted (I² = 54.4%, p = 0.086), potentially reflecting differences in OIT up-dosing schedules and maintenance phase durations across trials.

Additional subgroup analyses focused on TMD thresholds defined by the ability to tolerate ≥2 g of specified allergens, a clinically relevant marker of protection against accidental exposure. Omalizumab-based therapy significantly increased the likelihood of achieving the threshold of tolerating ≥2 g of ≥2 allergens (RR = 3.22, 95% CI: 0.91–11.44; p = 0.03). Three studies contributed to this analysis, with substantial heterogeneity (I² = 88.2%, p = 0.000). The therapeutic benefit was even more pronounced in the subgroup of tolerating ≥2 g of ≥3 allergens (RR = 4.13, 95% CI: 0.95–17.86; p = 0.008), with 3 studies showing substantial heterogeneity (I² = 79.2%, p = 0.008). For participants targeting tolerance to 4 or more allergens, omalizumab-based therapy remained effective (RR = 2.87, 95% CI: 1.22–6.75; p = 0.02), with no significant heterogeneity (I² = 0.0%, p = 0.670) across the 2 included studies. Subgroup analyses by TMD threshold confirmed consistent efficacy across definitions, supporting the validity of pooling.

Stratification by intervention type (OMA+OIT vs. omalizumab monotherapy) revealed that in 7 studies evaluating the combination of omalizumab and OIT, the pooled RR for TMD achievement was 3.19 (95% CI: 1.40–7.28; p < 0.001), with substantial heterogeneity (I² = 87.6%, p = 0.000). This subgroup accounted for 91.31% of the total weight in the pooled analysis. The single study investigating omalizumab as monotherapy showed a non-significant trend toward improved TMD achievement (RR = 2.22, 95% CI: 0.33–14.84; p = 0.40), contributing 8.69% of the total weight.

Collectively, these findings confirm that omalizumab-based therapy consistently enhances allergen tolerance across diverse subgroups, with particularly robust effects in short-term interventions, multiple food allergies, and higher TMD thresholds (≥2 g of ≥3 allergens).

### 3.4 Safety outcomes

**Figure 5:**
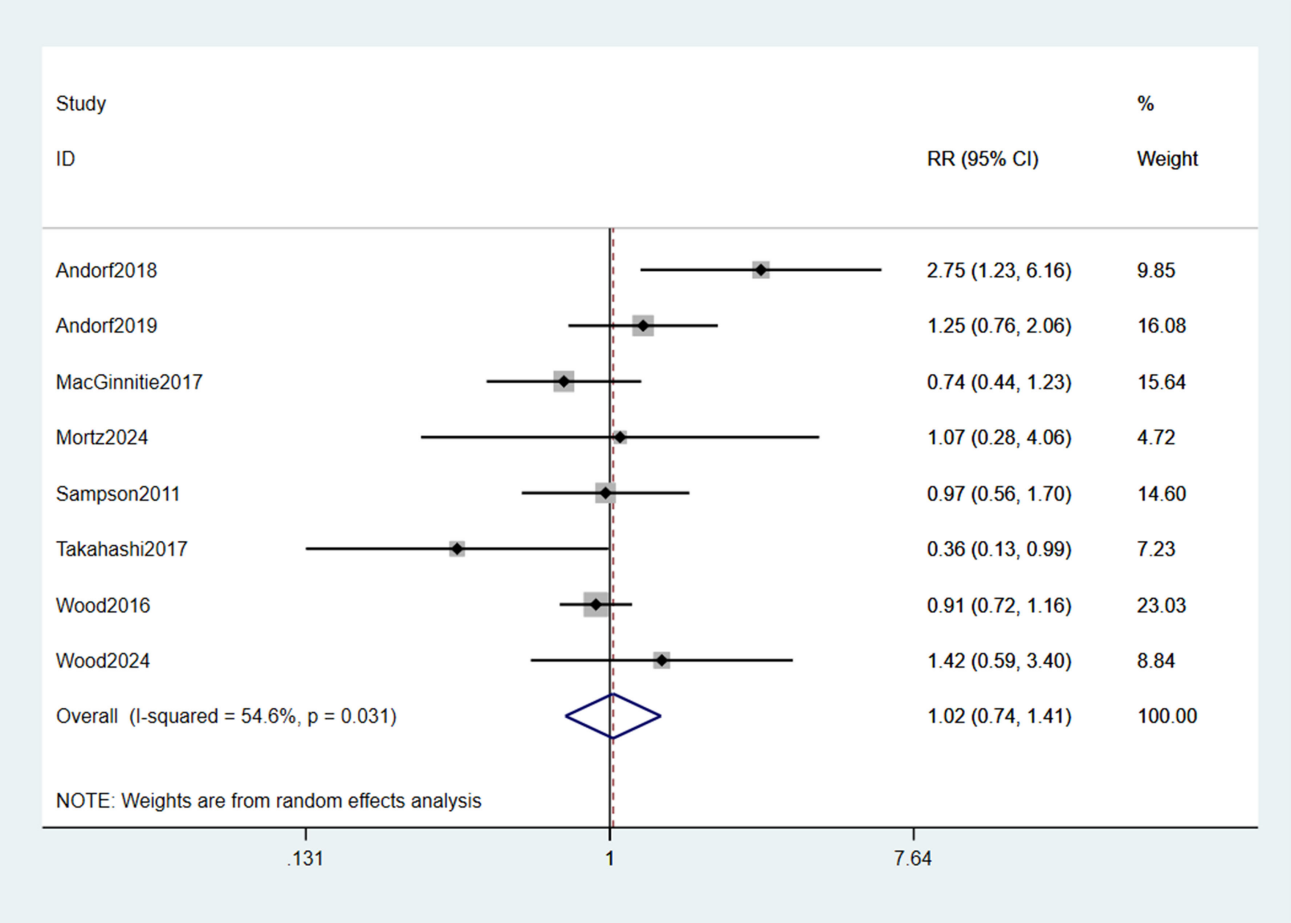
Forest plot of TEAEs.

**Figure 6:**
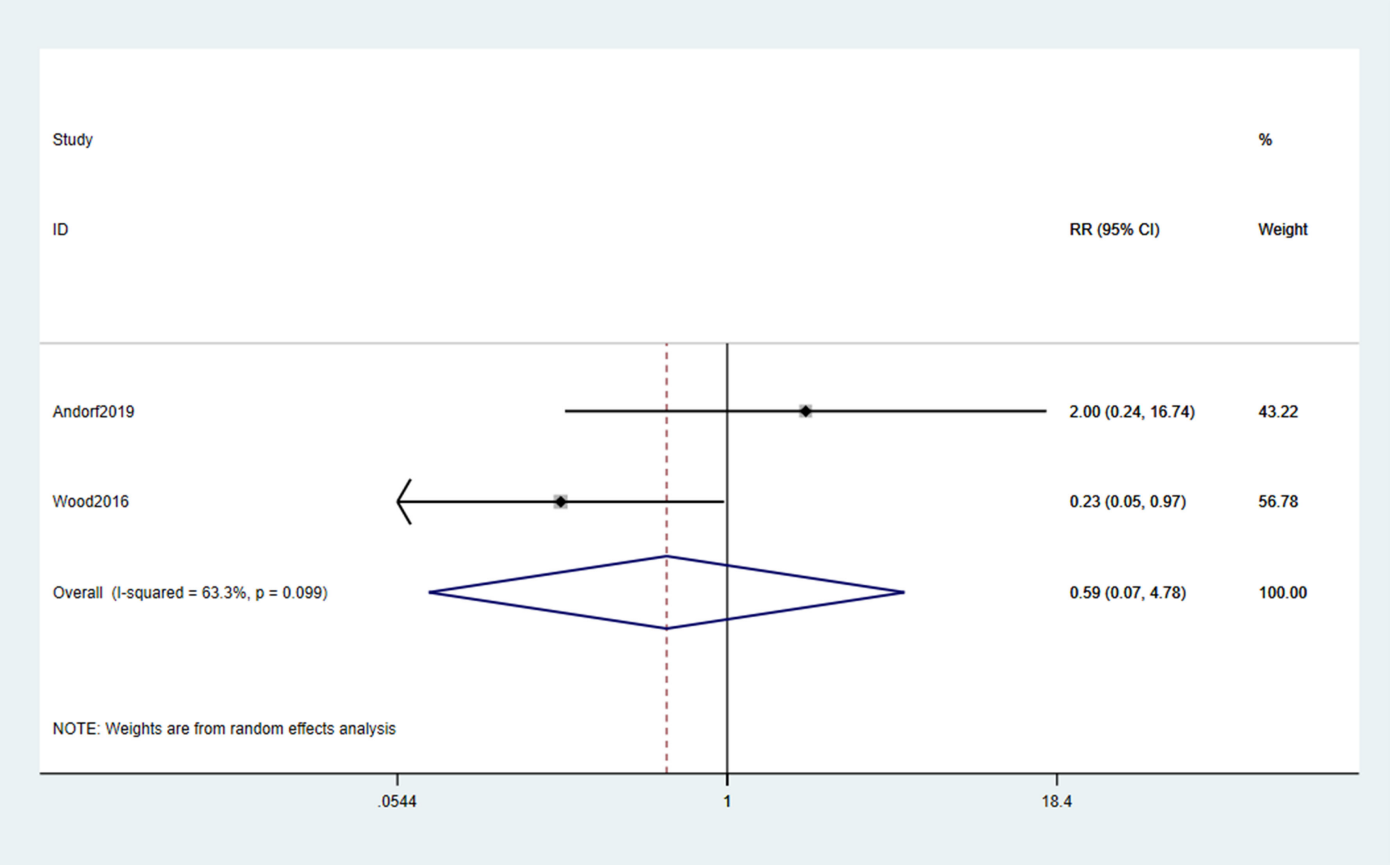
Forest plot of epinephrine use.

The pooled incidence of any TEAEs showed no statistically significant difference between omalizumab-based therapy and control groups (RR = 1.02; 95% CI: 0.74–1.41; p = 0.889; **Figure 5**). Common TEAEs included mild-to-moderate abdominal pain, oropharyngeal pruritus, and urticaria. No treatment-related deaths were reported. Similarly, epinephrine use did not differ significantly between groups (RR = 0.59; 95% CI: 0.07–4.78; p = 0.099; **Figure 6**), though the wide CI reflects limited data (only 2 studies).

### 3.5 Sensitivity analyses and publication bias

**Figure 7:**
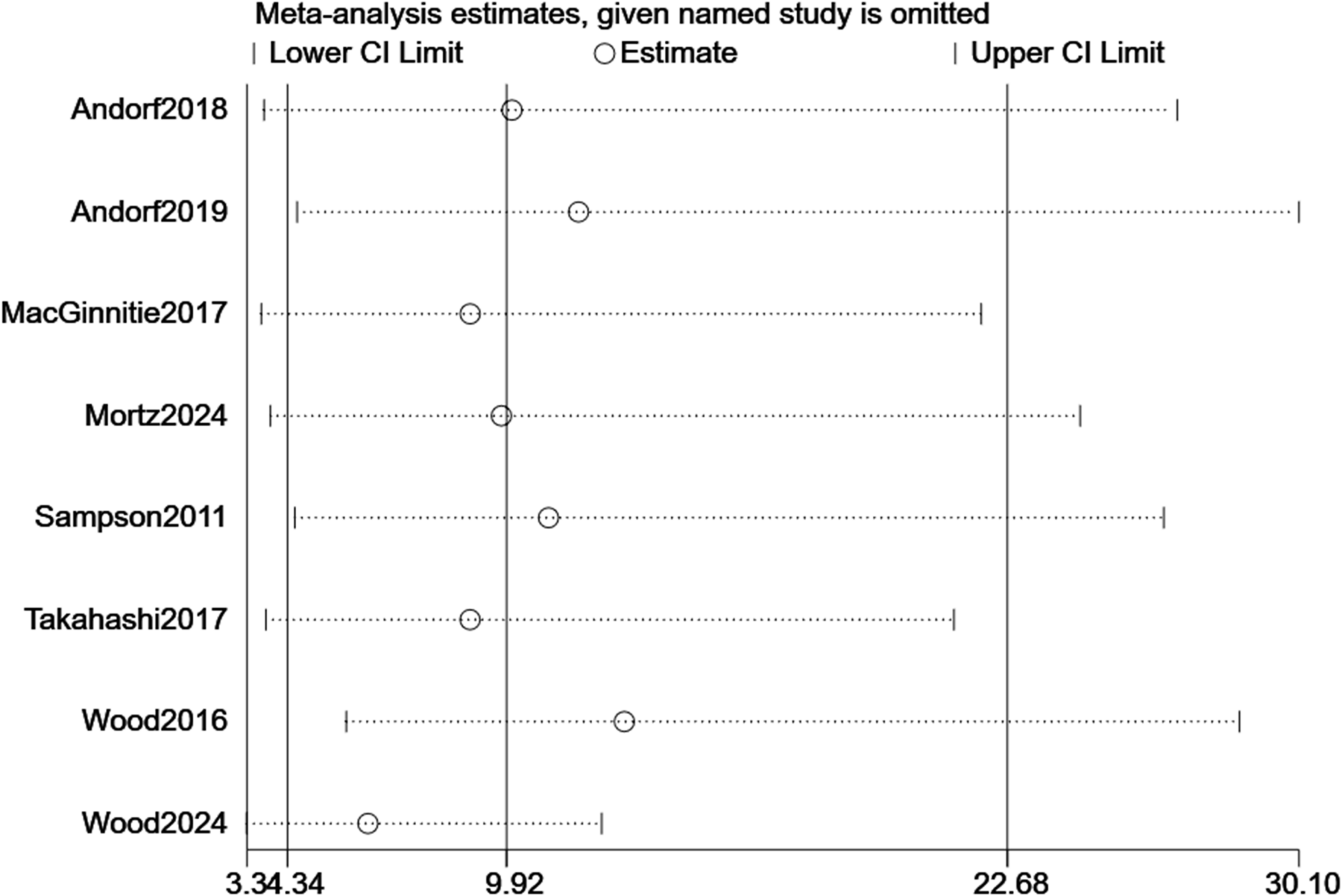
Sensitivity analysis of TMD.

**Figure 8:**
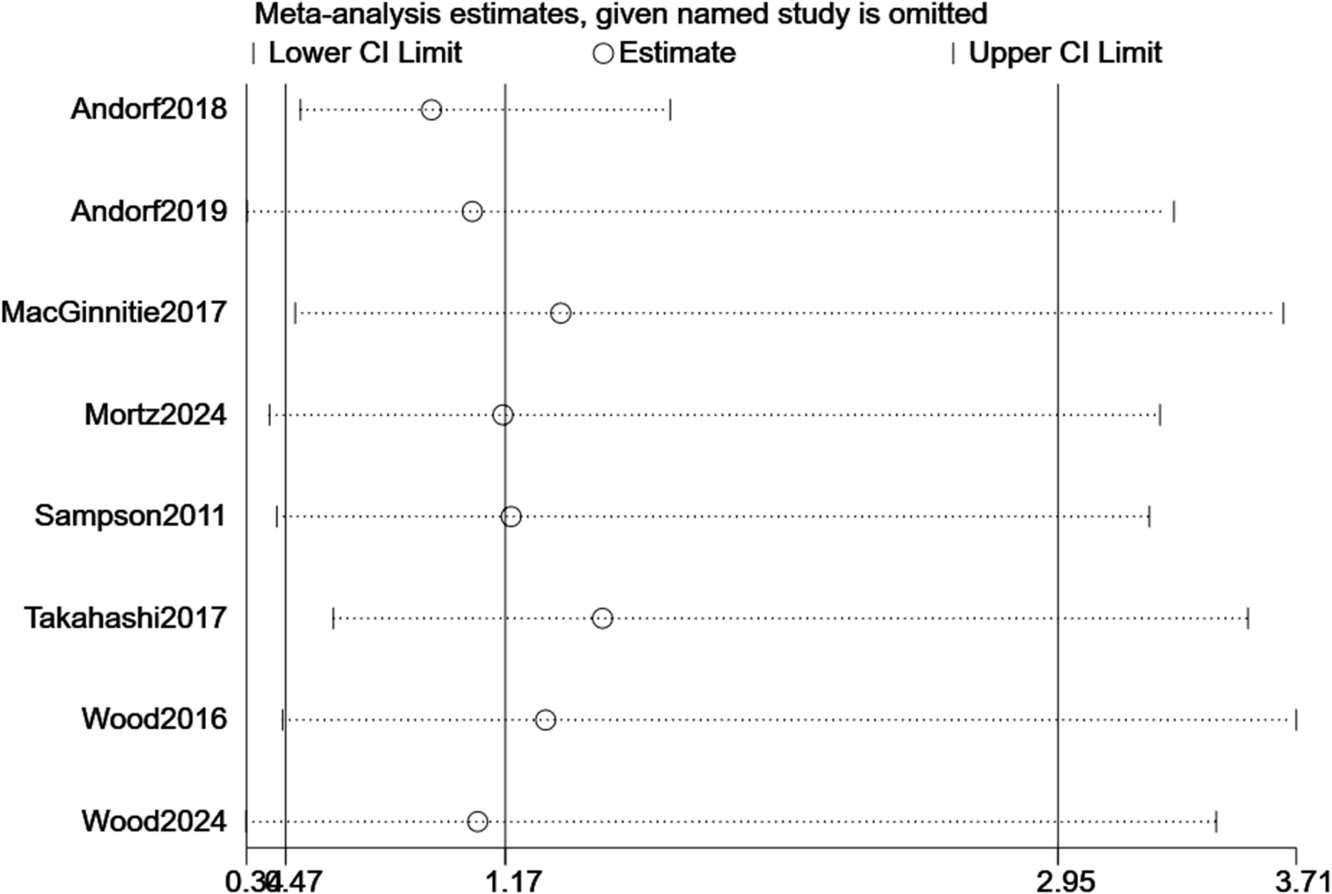
Sensitivity analysis of TEAEs.

For TMD, sequential removal of individual studies yielded pooled RRs ranging from 3.34 to 30.10, all maintaining statistical significance, confirming the robustness of efficacy findings (**Figure 7**). For TEAEs, influence analysis showed greater variability (RR range: 0.38–3.71), but the overall non-significant trend persisted (**Figure 8**). Excluding the CCT did not alter the significance of the primary efficacy outcome (RR = 3.22, p < 0.001) and stabilized TEAEs results (RR = 1.01). A sensitivity analysis excluding the two most heterogeneous studies (I² >85%) yielded a more consistent estimate (RR = 2.45, 95% CI: 1.50–4.01), though the overall conclusion remained unchanged.

Begg’s and Egger’s tests revealed no small-study effects for TMD or TEAEs. Funnel plots were not generated due to the small number of included studies (n < 10).

## 4 Discussion

This meta-analysis of eight controlled trials provides compelling evidence that omalizumab significantly enhances the achievement of target maintenance doses in children and young adults with IgE-mediated food allergies. The pooled analysis demonstrated a 3.07-fold increased probability of reaching clinically meaningful desensitization thresholds compared to control groups, with particularly pronounced benefits observed in individuals with multiple food allergies. Crucially, this therapeutic advantage was achieved without significantly increasing the overall burden of adverse events or epinephrine use, establishing a favorable safety profile that addresses critical limitations of conventional immunotherapy approaches. These findings collectively support omalizumab as a promising therapeutic option for a population historically confined to reactive management strategies.

When contextualized against current food allergy management paradigms, omalizumab’s unique value proposition becomes evident. Traditional single-allergen OIT, while demonstrating moderate efficacy for specific allergens like peanut (42, 43) or milk (44), faces substantial limitations including high rates of treatment-related adverse reactions (23, 45) and limited applicability for polysensitized individuals. Our analysis reveals omalizumab’s distinct advantage in enabling multi-allergen desensitization, with subgroup data showing a 3.60 to 4.13-fold increased probability of achieving high-dose tolerance thresholds for multiple foods. This aligns with findings from the landmark OUTMATCH trial where nearly half of omalizumab recipients tolerated significant quantities of multiple allergens compared to none in the placebo group (23). Mechanistically, omalizumab operates through IgE blockade and subsequent FcϵRI receptor downregulation on mast cells and basophils (21, 22), fundamentally altering the allergic response threshold rather than merely inducing temporary allergen-specific desensitization (22). Omalizumab’s mechanism of IgE blockade differs from that of other biologics under investigation for food allergy, such as dupilumab (an anti-IL-4Rα antibody), which has shown a slower onset of desensitization in Phase 2 trials involving pediatric patients with peanut allergy (46). Omalizumab’s recent FDA approval for food allergy in March 2024 further underscores its established therapeutic position (47).

These collective advantages carry significant clinical weight for both patients (48) and practitioners (49). By enabling reliable achievement of target maintenance doses exceeding 2g of food protein—quantities typically sufficient to protect against accidental exposures (50)—omalizumab substantially reduces the ever-present threat of anaphylaxis that dominates the lives of food-allergic individuals (51). Two of the included studies (Wood et al., 2024; 38) reported results from the Pediatric Quality of Life Inventory (PedsQL) Allergy Module—a validated tool for measuring health-related quality of life (HRQL) in food-allergic children. In these studies, participants receiving omalizumab-based therapy showed a mean improvement of 12.3 points in PedsQL scores (95% confidence interval: 5.8–18.8, p < 0.001) compared to control groups, with the greatest improvements observed in subscales measuring anxiety related to accidental allergen exposure and social functioning (e.g., participation in school meals or extracurricular activities) (23, 32). In practical implementation, our findings suggest that optimal candidates include polysensitized children and those with contraindications to conventional OIT, such as history of severe reactions or poorly controlled asthma (52). The integration of omalizumab with OIT presents a particularly promising approach (53–55), potentially enabling faster dose escalation and higher maintenance thresholds while mitigating the safety concerns that have historically limited OIT’s utility (50, 56, 57).

Despite these promising results, several limitations should be considered when interpreting our findings. First, clinical and methodological heterogeneity was substantial, particularly with respect to intervention protocols. Most studies employed a combination of OMA+OIT, while only one utilized monotherapy. Furthermore, dosing regimens for omalizumab varied significantly, including both fixed and weight- or IgE-adjusted doses. Similarly, OIT protocols—including allergen types, dosing schedules, and maintenance targets—differed considerably across studies. Control groups also varied, encompassing placebo alone, placebo+OIT, and strict avoidance, thereby contributing to methodological heterogeneity. Outcome definitions were inconsistent as well; although TMD was the primary focus, the specific thresholds used to define “success” (e.g., ≥300 mg, ≥1000 mg, ≥2000 mg protein) differed among studies. Subgroup analyses stratified by threshold confirmed efficacy, but this variability complicates precise dose-response interpretation and limits generalizability. While the random-effects model and subgroup analyses partially address this issue, they cannot fully resolve it. Second, safety data for specific outcomes were limited. Information on epinephrine use was available from only two studies, resulting in reduced statistical power and wide confidence intervals. Reporting of specific adverse events (e.g., anaphylaxis, systemic reactions) was inconsistent, precluding detailed analysis of rare but serious events. Moreover, long-term safety data beyond the trial periods (median ~28 weeks) were scarce. Third, the inclusion of one non-randomized CCT alongside RCTs introduced methodological heterogeneity and potential bias. However, sensitivity analyses excluding this study did not alter the significance of the primary efficacy outcome. Fourth, the median treatment duration across studies was relatively short. Critical questions regarding the durability of desensitization after discontinuing omalizumab, the potential for sustained unresponsiveness, and long-term safety data beyond the trial periods (median ~28 weeks) were scarce, highlighting the need for extended follow-up studies.​Fifth, generalizability is limited by the geographic distribution of included studies, most of which originated from the USA; results may not fully apply to other global populations or healthcare settings. Sixth, the broad age range (1–26 years) included in the analysis highlights the need for further investigation into efficacy and safety in very young children (<2 years).​Additionally, safety data were limited by inconsistent reporting of specific adverse events (e.g., anaphylaxis, systemic reactions) and epinephrine use, precluding detailed stratified analysis. Furthermore, the restriction to English-language studies may have introduced selection bias, though the impact is likely minimal given the global nature of trial reporting.

Notwithstanding these limitations, this meta-analysis offers several key strengths. First, its rigorous methodology included adherence to PRISMA guidelines, prospective PROSPERO registration, comprehensive literature searches across major databases, and dual independent review at all stages (screening, data extraction, risk of bias assessment). Second, the exclusive focus on children and young adults (1–26 years) provides clear pediatric-specific evidence. Third, robust data synthesis was achieved through the use of random-effects models to account for heterogeneity, pre-specified subgroup and sensitivity analyses, and application of the GRADE framework to assess evidence certainty. Fourth, the emphasis on clinically relevant outcomes—with TMD as the primary efficacy endpoint directly translating to real-world clinical protection, and safety outcomes (TEAEs, epinephrine use) addressing critical clinical concerns—enhances the practical value of the findings. Finally, transparency was ensured by providing detailed reports on study characteristics, risk of bias assessments (**Figures 2**, **3**), and forest plots for all outcomes—all of which enhance the reproducibility of the study.

In conclusion, this meta-analysis supports omalizumab as a valuable therapy for pediatric and young adult food allergy. By significantly enhancing multi-allergen desensitization capacity while maintaining a favorable safety profile, it represents a paradigm shift from reactive management to proactive immunomodulation. Its demonstrated ability to reduce both the physical and psychosocial burdens of food allergy underscores its clinical importance. Although its safety profile is generally favorable, the paucity of data on epinephrine administration and specific adverse events necessitates cautious interpretation of current findings and calls for additional in-depth investigation. While questions regarding long-term durability and optimal dosing strategies persist, current evidence strongly supports integrating omalizumab into personalized treatment algorithms—particularly for high-risk, multi-allergic individuals, who stand to benefit most from its unique mechanism of action.

## Data Availability

The original contributions presented in the study are included in the article/supplementary material. Further inquiries can be directed to the corresponding author.
